# Outcome of Patients with Locally Advanced Rectal Cancer Pursuing Non-Surgical Strategy in National Cancer Database

**DOI:** 10.3390/cancers16122194

**Published:** 2024-06-11

**Authors:** Hanna Kakish, Fasih A. Ahmed, Lee M. Ocuin, Jennifer L. Miller-Ocuin, Emily Steinhagen, Richard S. Hoehn, Amit Mahipal, Christopher W. Towe, Sakti Chakrabarti

**Affiliations:** 1 Department of Surgery, Division of Surgical Oncology, University Hospitals Cleveland Medical Center, Cleveland, OH 44106, USA; 2 Department of Surgery, University of Pennsylvania, Philadelphia, PA 19104, USA; 3 Department of Surgery, Division of Colorectal Surgery, University Hospitals Cleveland Medical Center, Cleveland, OH 44106, USA; 4 Department of Oncology, University Hospitals Seidman Cancer Center, Case Western Reserve University, Cleveland, OH 44106, USA; 5 Division of Thoracic and Esophageal Surgery, Department of Surgery, Case Western Reserve School of Medicine, University Hospitals Cleveland Medical Center, Cleveland, OH 44106, USA

**Keywords:** locally advanced rectal cancer, surgery, non-operative management, overall survival

## Abstract

**Simple Summary:**

The current study examined the outcomes of patients with locally advanced rectal cancer (LARC) who underwent non-operative management (NOM) instead of surgery in routine clinical practice. Using data from the National Cancer Database, we found that those who did not undergo surgery had lower survival rates than those who had surgical interventions. Specifically, patients with high-risk features, for example, patients with T4 tumors, had much more inferior survival with NOM than with surgery. These findings suggest that opting for NOM might result in worse survival outcomes, underscoring the need for further research to confirm these results.

**Abstract:**

Background: Survival data on patients with locally advanced rectal cancer (LARC) undergoing non-operative management (NOM) in a real-world setting are lacking. Methods: We analyzed LARC patients from the National Cancer Database with the following features: treated between 2010 and 2020, age 18–65 years, Charlson comorbidity index (CCI) ≤ 1, received neoadjuvant multiagent chemotherapy plus radiation ≥ 45 Gray, and underwent surgery or NOM. Patients were stratified into two groups: (A) clinical T1-3 tumors with positive nodes (cT1-3N+) and (B) clinical T4 tumors, N+/− (cT4N+/−). We performed a comparative analysis of overall survival (OS) with NOM versus surgery by the Kaplan–Meier method and propensity score matching. Additionally, a multivariable analysis explored the association between NOM and OS. Results: NOM exhibited significantly lower OS than surgery in both groups. In cT1-3N+ patients, NOM resulted in a 5-year OS of 73.9% (95% confidence interval [CI] = 69.7–77.6%) versus 84.5% (95% CI = 83.6–85.3%) with surgery (*p* < 0.001). In the cT4N+/− group, NOM yielded a 5-year OS of 44.5% (95% CI = 37.0–51.8%) versus 72.5% (95% CI = 69.9–74.8%) with surgery (*p* < 0.001). Propensity score matching and multivariable analyses revealed similar conclusions. Conclusion: Patients with LARC undergoing NOM versus surgery in real-world settings appear to have inferior survival.

## 1. Introduction

Locally advanced rectal cancer (LARC) that encompasses stage II (T3-4, node-negative) and stage III (node-positive) disease represents a distinct subset of rectal cancer with unique treatment considerations. Presently, the preferred approach for patients with LARC includes total neoadjuvant therapy (TNT), consisting of sequential administration of concurrent chemoradiation (CRT) and multiagent chemotherapy, followed by surgical intervention or non-operative management (NOM) in patients achieving clinical complete response (cCR) [1]. The upfront TNT approach has recently superseded the earlier sequence of CRT followed by surgery and risk-adapted adjuvant chemotherapy [2,3]. Two pivotal phase III studies, RAPIDO [4] and PRODIGE23 [5], have solidified the standing of TNT in LARC treatment protocols. A notable benefit of upfront TNT, as evidenced in several large studies [6,7,8,9,10,11,12,13,14,15,16], including the Organ Preservation in Patients With Rectal Adenocarcinoma (OPRA) study [17,18], is the enhanced potential for organ preservation. The organ preservation approach aims to mitigate various post-surgical complications that can affect the quality of life, causing, for example, bowel, urinary, and sexual dysfunction [19,20].

Organ preservation for patients with LARC is gaining momentum in routine clinical practice [21]. Despite accumulating data supporting the role of a non-operative approach, it is noteworthy that mature data are lacking to ensure that survival is equivalent to standard operative management in all subsets of patients with LARC. Furthermore, a randomized trial does not exist to date in which patients with LARC achieving cCR after TNT were assigned to surgery versus NOM. The question surrounding the long-term efficacy and safety of NOM is more relevant for patients at a higher risk of local and systemic recurrence [1]. To address this concern, we conducted a retrospective analysis utilizing real-world data sourced from the National Cancer Database (NCDB) of the United States. Our analysis compared survival outcomes between cohorts undergoing NOM versus surgery. This analysis aims to provoke inquiry into the appropriateness of the widespread adoption of the NOM strategy in routine clinical practice for patients with LARC, particularly among those deemed at higher risk of both local and systemic recurrences.

## 2. Methods

### 2.1. Data Source

We sourced data from the NCDB. The NCDB is a comprehensive, nationwide cancer registry that operates under the joint sponsorship of the American Cancer Society and the American College of Surgeons Commission on Cancer (CoC). The database is maintained by trained tumor registrars who meticulously collect demographic and clinical data pertaining to all cancer cases diagnosed within their respective healthcare facilities, adhering to rigorously standardized protocols. The NCDB encompasses a vast dataset, encompassing more than 70% of the cancer cases diagnosed across the US, drawing data from a network of over 1500 institutions. It is important to note that the data within the NCDB are rigorously de-identified, rendering it exempt from the requirements of institutional review board approval and patient informed consent. Furthermore, we adhered to the best practices for utilizing the NCDB [22] and the Strengthening the Reporting of Observational Studies in Epidemiology (STROBE) reporting guideline [23].

### 2.2. Study Population

**Figure 1:**
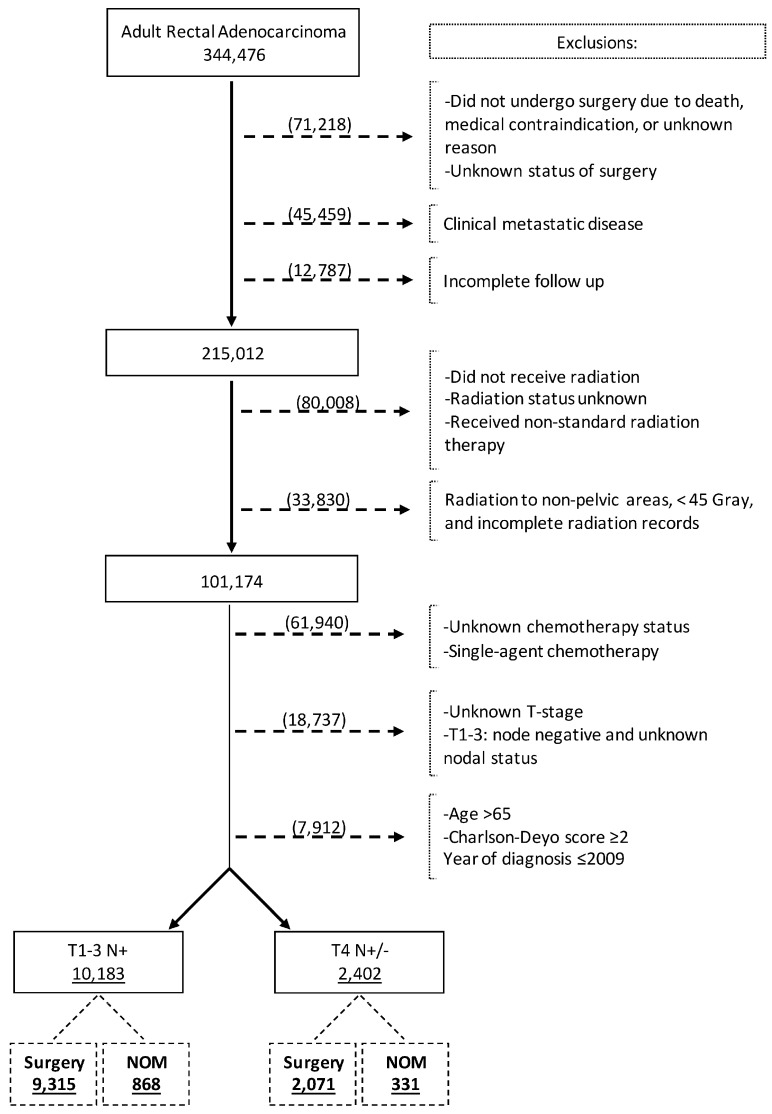
Flow diagram depicting patient selection process.

We reviewed the NCDB Participant User Files from 2010 to 2020 for adult patients (aged 18–65 years) diagnosed with rectal adenocarcinoma using the International Classification of Diseases for Oncology disease topography code for rectum (C209) and Histology codes of adenocarcinoma (8000, 8010, 8140, 8144, 8210, 8211, 8213, 8220, 8221, 8255, 8260, 8261, 8262, 8263, 8480, and 8481) [24]. The patient selection process is outlined in Figure 1. Patients were selected based on the following inclusion criteria: 1. age 18–65 years, 2. Charlson comorbidity index (CCI) 0 or 1, 3. clinical stage II and III rectal cancer, 4. received neoadjuvant multiagent chemotherapy, 5. received at least 45 Gray of neoadjuvant pelvic radiation therapy, and 6. underwent rectal cancer surgery (surgery group) or did not undergo surgery (NOM group). Patients not undergoing surgery because of death, medical contraindication, or unknown reasons were excluded. Other exclusion criteria were as follows: 1. clinically stage IV disease at diagnosis, 2. incomplete follow-up data, 3. unknown surgery status, 4. unknown status of radiotherapy or incomplete radiation records, 5. unknown status of chemotherapy, 6. unknown T-stage, and 7. unknown or negative nodal status. The patient selection criteria were chosen to reflect the patient population that pursued the NOM strategy following achieving cCR.

The final analysis consisted of two study cohorts: A. patients with clinical T1-3 and node-positive (N+) LARC (cT1-3N+ cohort) and B. patients with clinical T4 and N+ or node-negative (N−) LARC (cT4 N+/− cohort). For the survival analysis, each cohort had two subsets: patients who underwent NOM and patients undergoing surgery. Data analysis took place between January and April of 2024.

### 2.3. Variables and Outcomes Measures

The following patient-level data were collected: age, gender (male, female), race (White, Black, Asian, Hispanic, and others/unknown), CCI (0, 1, 2, ≥3), insurance status (not insured, private insurance, Medicaid, Medicare, and others/unknown), T stage, radiation dose (≤5040 cGy vs. >5040 cGy), and the year of diagnosis. In addition, data on treatment facility type were collected (community cancer program, comprehensive community cancer program, academic cancer program, and integrated network cancer program). The primary outcome measure of this study was overall survival (OS). We compared the survival of LARC patients undergoing NOM versus surgery in cohorts A and B. Survival time was calculated in months from diagnosis to last contact or death. Patients lost to follow-up after treatment were censored at the date of their last recorded follow-up.

### 2.4. Statistical Analysis

To describe the baseline characteristics of patients, we employed descriptive statistics. Demographics, disease-related features, and facility attributes of the patients were analyzed using frequencies and percentages. Differences in the distribution of the patient characteristics were compared using the Pearson chi-square test. Kaplan–Meier survival estimates were used to evaluate OS and log-rank testing was used for survival comparisons between patient groups. Multivariable analyses were performed using Cox regression analysis to determine the impact of various factors on OS and, accordingly, hazard ratios (HRs) with corresponding 95% confidence interval (CI) values were generated. The factors included in the multivariable analyses were age, sex, race, CCI, NOM versus surgical resection, insurance status, facility type, and radiation dose (<50.4, 50.4–54, or >54 Gy). All statistical calculations were performed using STATA, version 17.0 (StataCorp). A two-sided α < 0.05 was considered statistically significant.

A propensity score matching analysis was performed to mitigate the potential impact of confounding variables, thereby enhancing the ability to glean valuable insights into the long-term survival outcomes associated with NOM versus surgical management in the study subsets. Using the nearest neighbor method, the propensity scores for the NOM and surgical groups were calculated through logistic regression, and the groups were matched with a caliper width equal to 0.1 of the standard deviation of the logit of propensity score and a critical stipulation of “without replacement” to ensure the fidelity of the matched cohorts [25]. The factors considered in this propensity matching included age, sex, race, CCI, insurance status, radiation dose, facility type, and nodal status (all these factors achieved a standardized mean difference of less than 0.1 and were deemed adequately matched). It is important to note that only those variables achieving a standardized mean difference of less than 0.1 were deemed adequately matched. To gauge the effectiveness of the propensity matching, we examined changes in standardized bias, aiming to bring it within acceptable thresholds. Additionally, a Mahalonobis distance graph was plotted, serving as a visual representation of the impact of propensity matching on the distribution of covariates. The survival outcomes between the NOM and the surgically managed cohorts were compared using Kaplan–Meier survival curves [26].

## 3. Results

### 3.1. Baseline Characteristics of the Study Cohort

The database search identified 344,476 patients with rectal adenocarcinoma, of whom 12,585 fulfilled the inclusion criteria and were included in the analysis (Figure 1). The cT1-3 N+ and cT4N+/− study cohorts comprised 10,183 (80.9%) and 2402 (19.1%) patients, respectively. In the whole study cohort, the median age at diagnosis was 53 years (Interquartile range [IQR] = 47–59), 61.8% were male, and 76.6% were Non-Hispanic White (Table 1). Overall, 71.8% of patients had private insurance and 35.5% were treated at academic centers. Of patients in the cT1-3 N+ cohort, 868 (8.5%) underwent NOM. Among the patients with cT4N+/− disease, 331 (13.8%) underwent NOM. Patients in the NOM group were more likely to receive a radiation dose greater than 5040 cGy.

### 3.2. Survival Analysis

**Figure 2:**
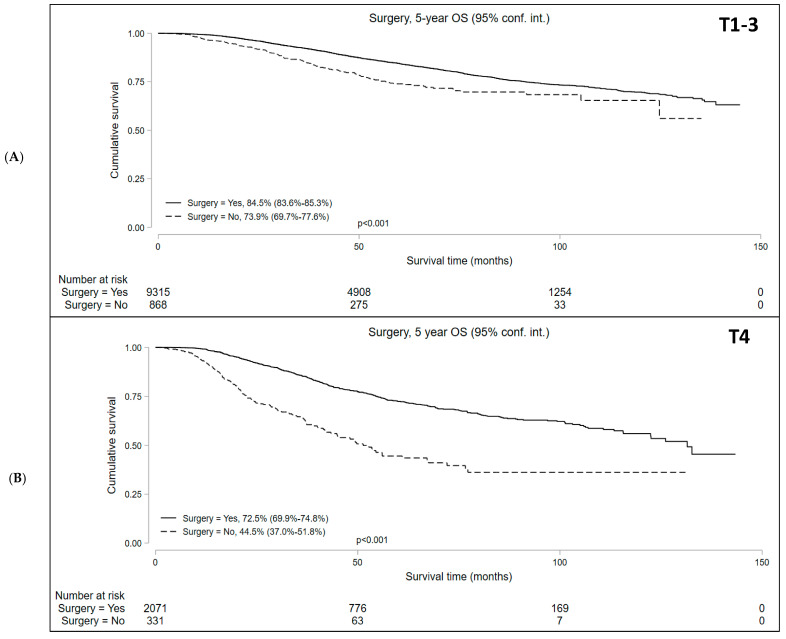
Overall survival of patients with locally advanced rectal cancer undergoing surgery versus non-operative management (NOM) in the (**A**) T1-3N+ and (**B**) T4N+/− cohorts.

**Figure 3:**
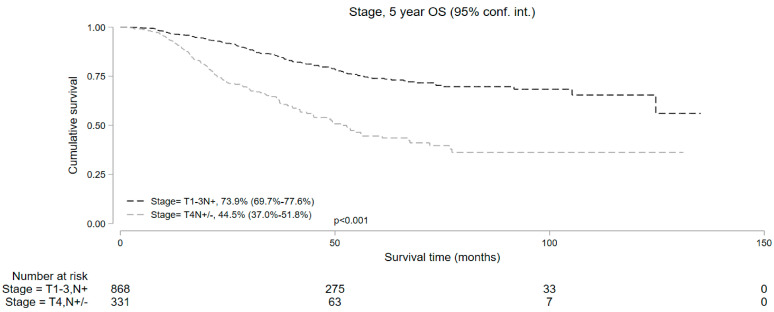
Five-year overall survival of patients with T1-3N+ and T4N+/− locally advanced rectal cancer undergoing non-operative management.

Median follow-up was 50.9 and 37.7 months for cT1-3 N+ and cT4 N+/− cohorts, respectively. Kaplan–Meier survival analysis showed that the median OS in patients who underwent NOM was significantly lower compared to those who underwent surgery in both study subsets. In the cT1-3 N+ cohort, the 5-year OS with NOM and surgery was 73.9% (95% CI = 69.7–77.6%) and 84.5% (95% CI = 83.6–85.3%), respectively (*p* < 0.001). In the cT4 N+/− cohort, the 5-year OS was 44.5% (95% CI = 37.0–51.8%) and 72.5% (95% CI = 69.9–74.8%) in the NOM and surgery groups, respectively (*p* < 0.001) (Figure 2). In addition, among the patients undergoing NOM, the OS was superior in the cT1-3 N+ group than the cT4 N+/− group (*p* < 0.001) (Figure 3).

We performed multivariable analyses to evaluate the impact of NOM on OS in each study cohort (Table 2). Non-operative management was associated with significantly inferior OS in both cohorts (HR = 1.65, 95% CI = 1.40–1.94 for cT1-3 N+ group and HR = 2.73, 95% CI = 2.24–3.32 for cT4 N+/− group).

### 3.3. Propensity Matched Comparison of Surgery and NOM

**Figure 4:**
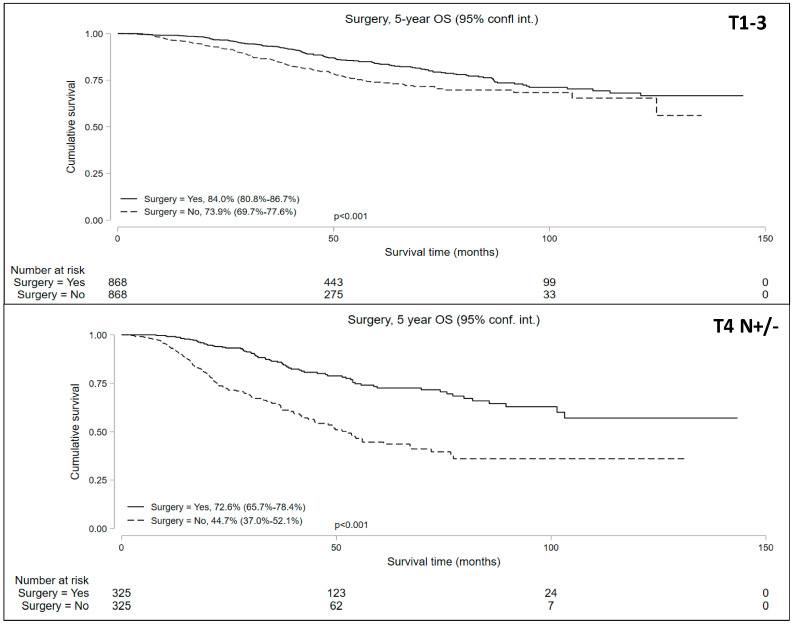
Five-year overall survival of patients with locally advanced rectal cancer undergoing non-operative management (NOM) versus surgery in the matched cohorts.

The propensity-matched analysis reduced bias between the groups (Supplemental Table S1, Supplemental Figure S1) and resulted in well-balanced cohorts: 868 matched pairs in the cT1-T3N+ patients undergoing NOM vs. surgery and 325 matched pairs in the cT4N+/− patients undergoing NOM vs. surgery. The median follow-up periods were 42.8 and 33.4 months for the cT1-3 N+ and cT4 N+/− cohorts, respectively. Univariable analysis of the matched pairs showed a significantly lower 5-year OS in patients who underwent NOM in both cohorts (Figure 4). Five-year OS in the NOM vs. surgery groups in the propensity-matched cohorts was 73.9% (95% CI = 69.7–77.6%) vs. 84.0% (95% CI = 80.8–86.7%) for the cT1-3 N+ cohort and 44.7% (95% CI = 37.9–52.1%) vs. 72.6% (95% CI = 65.7–78.4%) for the cT4 N+/− cohort.

## 4. Discussion

The primary objective of our study was to evaluate survival in patients with LARC undergoing NOM versus surgery in a real-world setting. This study particularly emphasized the subset of patients with cT4 tumors, given their increased risk of local and distant recurrences impacting survival. Our findings indicate a significantly lower OS in patients with cT4 tumors receiving NOM than those who underwent surgery. In patients with cT1-3N+ tumors, NOM was similarly associated with a decrease in OS compared to surgery, albeit the magnitude of this difference was less pronounced. Notably, these outcomes were consistent in multivariable analyses adjusted for established prognostic factors, as well as in propensity score analyses. To our knowledge, this represents the first analysis utilizing NCDB data that compare the survival of patients with LARC undergoing NOM versus surgery.

An overarching question is whether the patients included in the NOM group in the current analysis genuinely reflected the patients who pursued a non-operative approach after achieving cCR following TNT. This query arises due to NCDB’s limitations, which lack comprehensive data on responses to TNT. However, our patient selection criteria were meticulously crafted to include younger patients (<65 years) with minimal comorbidities (CCI, 0 or 1) who received multiagent chemotherapy and a pelvic radiation dose ≥ 45 Gy to ensure the exclusion of patients who did not undergo surgery for being surgically unfit. Our patient selection criteria included patients who typically pursued NOM after achieving cCR with TNT. It is plausible that a subset of patients within this group may have been unable to pursue surgery due to complications arising from TNT or because of disease progression. However, the likelihood of this patient subgroup constituting a substantial proportion is quite low. This assertion is supported by several prospective studies that have reported that fewer than 10% of LARC patients were unable to undergo surgery following the completion of neoadjuvant therapy [4,5,27]. Furthermore, the temporal trend of treatment patterns between 2010 and 2020 was consistent: the highest proportion of patients undergoing NOM in our study were treated in 2015 and later (Table 1). This observation is congruent with the recognized trend of increasing the utilization of NOM as a viable treatment strategy in recent years [21], supporting the idea that most patients in our NOM cohort pursued a non-operative strategy after achieving cCR.

The published literature suggests that distant metastasis (DM) may contribute to poorer survival in patients with LARC pursuing NOM. The incorporation of neoadjuvant CRT and total mesorectal excision (TME) in the treatment of LARC reduced the pelvic recurrence rates to less than 10%; however, the DM rate and OS did not show significant improvement, remaining at approximately 30% and 65%, respectively [28,29,30]. A large volume of data demonstrated that NOM is associated with local regrowth following initial complete clinical response (cCR) in 25–30% of patients, predominantly occurring within the first two years of follow-up [16,18,31]. Local regrowth is a known risk factor for DM, as demonstrated in several studies [14,31], which likely adversely impacts survival.

The risk of DM in patients with LARC undergoing CRT followed by surgery prompted the design of TNT, wherein systemic chemotherapy is administered before surgery, either before or after CRT. Two pivotal phase III trials, RAPIDO [4] and PRODIGE23 [5], examined the impact of TNT on the incidence of DM. Both studies demonstrated a statistically significant reduction in DM rates in the TNT cohorts: 20% versus 27% in RAPIDO [4] and 17% versus 25% in PRODIGE23 [5]. Over the last decade, two TNT protocols have emerged: induction chemotherapy followed by CRT (CT-CRT sequence) and CRT followed by consolidation chemotherapy (CRT-CT sequence). However, an optimal TNT sequence remains debatable [32]. Encouraged by the OPRA trial results, the NOM approach typically utilizes the CRT-CT sequence since the CRT-CT arm in the OPRA study arm demonstrated the highest rate of TME-free survival [17,18]. The CRT-first approach delays the initiation of systemic chemotherapy by several months. The delay in administering systemic (adjuvant) chemotherapy is a recognized risk factor for DM with adverse survival outcomes in patients with resected early-stage colon cancer, as demonstrated in multiple studies [33,34,35,36]. A posthoc analysis of the RAPIDO trial revealed a significantly lower cumulative probability of DM at 5 years in the experimental arm, where systemic chemotherapy was administered early, immediately after the short-course radiation, compared to the arm receiving chemotherapy after surgery: 23% [95% CI, 19–27] vs. 30% [95% CI, 26–35], with a hazard ratio (HR) of 0.72 [95% CI, 0.56–0.93], *p* = 0.011 [37]. As the CRT-CT sequence is increasingly being utilized to achieve cCR and pursue NOM, it is likely that DM is playing a critical role in adversely affecting the survival of patients undergoing NOM. The apprehension regarding the delay in administering systemic chemotherapy is readily evident in the recent study designs, particularly in two studies designed by the German Rectal Cancer Study Group, namely, CAO/ARO/AIO-12 [38] and the ongoing ACO/ARO/AIO-18.1 (NCT04246684). In these trials, patients assigned to the long-course CRT arm received multiagent chemotherapy (oxaliplatin and 5-fluorouracil) concurrently with radiation therapy. Notably, the incorporation of multiagent chemotherapy into the regimen of long-course CRT has demonstrated improved disease-free survival and pCR rate compared with fluorouracil-based CRT in the CAO/ARO/AIO-04 study [39].

The significantly reduced OS observed in patients who underwent NOM instead of surgery raises concerns. It is especially disconcerting given that the NOM approach is typically chosen for a specific subgroup of ‘biologically well-behaving’ patients who achieve cCR. In the current analysis, the NOM group was compared to a heterogeneous cohort of patients who underwent surgery without consideration of the depth of response to TNT. In other words, the surgical group included patients across a spectrum of response levels and was not restricted to the carefully selected ‘biologically well-behaving’ patients. Nevertheless, despite these differences, the NOM group exhibited significantly inferior survival, which raises concern. Age and comorbidity are unlikely to be the sole explanation for this difference, as the difference in the median age in the NOM vs. surgery group (cT1-3N+: 56 vs. 61 years and cT4N+/−: 57 vs. 60 years) was minor, and there were no significant differences in the comorbidity between the groups.

The publication of the OPRA trial results has provided additional momentum towards the adoption of NOM in patients with LARC [17,18]. This phase II randomized trial enrolled 324 patients with stage II/III rectal cancer who were assigned to either an induction multiagent chemotherapy followed by CRT (CT-CRT arm, n = 158) or CRT followed by consolidation chemotherapy (CRT-CT arm, n = 166). Patients who achieved cCR or near-cCR post-treatment were considered for NOM. The primary endpoint was disease-free survival (DFS) and the secondary endpoint was TME-free survival. After a median follow-up of 5.1 years, a significantly higher TME-free survival rate was observed in the CRT-CT arm: 54% (95% CI, 46 to 62) versus 39% (95% CI, 32 to 48), *p* = 0.012. The 5-year DFS rates were comparable between the two arms (71% vs. 69%, *p* = 0.68), as were the rates of DM-free survival. The observed low DM rate and the absence of an increase in DM in the CRT-CT arm are likely linked to the baseline tumor characteristics. In the OPRA trial, only a minority of patients had cT4 tumors (11% in the CRT-CT arm and 15% in the CT-CRT arm), and the extent of lymph node involvement (cN1 vs. cN2) was not detailed. Notably, none of the patients exhibited well-known risk factors for DM, including extramural venous invasion (EMVI) or mesorectal fascia (MRF) involvement, and less than 5% of patients had high-grade tumor histology. Therefore, it is critical to consider that patients with LARC exhibiting high-risk features, such as cT4 tumors or extensive lymph node involvement, might not be ideal candidates for NOM. The ongoing ACO/ARO/AIO-18.1 study, a phase III randomized study that assigns patients with LARC, including high-risk patients, to either short-course radiotherapy or long-course CRT followed by consolidation chemotherapy in both arms and then either surgery or watch-and-wait approach, is expected to provide valuable insights into the efficacy and safety of the non-operative strategy in patients with high-risk LARC.

Approximately 10% of patients with localized rectal cancer present with cT4 tumors [40]. The adverse prognostic impact of T4 tumors in patients with early-stage colorectal cancer is well-established [40,41,42,43,44]. Notably, in the case of early-stage resected colon cancer, the T4 tumor stage has been identified as a significant independent risk factor for distant recurrence, irrespective of the overall tumor–node–metastasis (TNM) stage, in numerous studies [41,42]. Consequently, all national clinical guidelines advocate for the use of adjuvant chemotherapy to mitigate the risk of systemic recurrence in individuals with resected early-stage colon cancer harboring T4 tumors [45,46,47]. A similar adverse prognostic impact of T4 tumor has been observed in patients with LARC [40,48]. In the context of LARC, the risk of distant recurrence significantly outweighs the risk of local recurrence, which contributes substantially to cancer-related morbidity and mortality [30,40,49]. A retrospective study demonstrated that early tumor regrowth following an initial cCR with neoadjuvant therapy is notably more frequent among patients with distal rectal cancer characterized by cT3/4 tumors compared to those with cT2 tumors (30% vs. 3%, *p* = 0.007) [50]. These findings further support our concern that patients with LARC harboring cT4 tumors might experience a significant reduction in OS with NOM compared to patients who undergo surgery, underscoring the importance of an appropriate patient selection strategy for NOM.

It is imperative to acknowledge several limitations of the current study. Primarily, this analysis is retrospective, and, as such, it inherits the customary constraints and biases associated with studies of this type. Specifically, the NCDB lacks the granularity of detailed individual-level data, necessitating caution in interpreting the data. It is crucial to recognize the potential for data entry errors and miscoding, although it is noteworthy that the CoC has implemented rigorous quality control measures to mitigate some of these concerns [51]. Another notable limitation stems from the NCDB’s lack of comprehensive information regarding chemotherapy specifics, including details such as duration and type. Furthermore, a significant limitation arises from the challenge of distinguishing between patients who did not undergo surgery because they achieved cCR and those who were unable to undergo surgery due to disease progression or treatment-related toxicities, rendering them inoperable. To mitigate potential bias stemming from the latter group, we excluded patients who did not proceed with surgery due to reasons such as death, medical comorbidities, or unknown causes. Finally, the database primarily focuses on overall survival as the sole long-term outcome metric, limiting the scope of our analysis. However, despite these acknowledged limitations, the striking disparity in survival outcomes between patients undergoing NOM versus surgery is a compelling observation. This discrepancy prompts us to exercise caution before advocating for a universal application of NOM in all patients with LARC, regardless of their risk profile. These data also support a prospective investigation of the long-term efficacy and safety of NOM in high-risk patients, including patients with cT4 tumors.

A critical question at this juncture is how to address the concerns raised by the current dataset. As previously indicated, an appropriate clinical trial design to evaluate the long-term efficacy and safety of the NOM strategy would be a randomized, phase III trial. In this trial, patients with LARC achieving cCR would be randomized to either standard surgery or NOM, with a well-balanced distribution of risk characteristics in each arm. The trial design should incorporate a preplanned subset analysis for high-risk patients and include robust endpoints such as 3-year or 5-year disease-free survival (DFS), overall survival (OS), local and systemic recurrence rates, and quality of life measures, including for bowel, urinary, and sexual function. Additionally, uniform criteria for determining cCR should be applied to all patients. A post-hoc analysis of the genomic profiles of patients achieving long-term survival compared to those experiencing cancer relapse would further enhance our understanding and aid in the precise selection of patients for NOM.

In summary, the current analysis underscores the importance of carefully selecting patients when considering organ preservation for patients with LARC. It specifically advises against the indiscriminate adoption of the non-operative strategy in patients who exhibit a higher risk of systemic failure. The results presented in this study are hypothesis-generating and strongly advocate for a randomized controlled trial targeting high-risk LARC patients who have achieved cCR following multimodality neoadjuvant therapy, comparing survival outcomes between the non-operative strategy and surgery.

## Figures and Tables

**Table 1:** Characteristics of patients with locally advanced rectal cancer undergoing non-operative management (NOM) versus surgery.

Factor	T1-3 N+, n (%)			T4 N+/−, n (%)		
	Surgery	NOM	*p*-Value *	Surgery	NOM	*p*-Value *
Total	9315	868		2071	331	
Age, Median (Interquartile range)	53 (47, 59)	53 (46, 60)	0.065	54 (47, 60)	55 (47, 60)	0.720
Sex						
Male	5815 (62.4%)	581 (66.9%)	0.009	1177 (56.8%)	200 (60.4%)	0.220
Female	3500 (37.6%)	287 (33.1%)		894 (43.2%)	131 (39.6%)	
Race						
Non-Hispanic White	7226 (77.6%)	659 (75.9%)	0.220	1553 (75.0%)	206 (62.2%)	<0.001
Non-Hispanic Black	664 (7.1%)	59 (6.8%)		154 (7.4%)	51 (15.4%)	
Asian	429 (4.6%)	35 (4.0%)		93 (4.5%)	20 (6.0%)	
Hispanic	690 (7.4%)	79 (9.1%)		214 (10.3%)	49 (14.8%)	
Others/Unknown	306 (3.3%)	36 (4.1%)		57 (2.8%)	5 (1.5%)	
Charlson Comorbidity Index						
0	8118 (87.1%)	766 (88.2%)	0.350	1742 (84.1%)	304 (91.8%)	<0.001
1	1197 (12.9%)	102 (11.8%)		329 (15.9%)	27 (8.2%)	
Radiation dose						
≤5040	8886 (95.4%)	801 (92.3%)	<0.001	1943 (93.8%)	290 (87.6%)	<0.001
>5040	429 (4.6%)	67 (7.7%)		128 (6.2%)	41 (12.4%)	
Cancer Program Type						
Community	456 (4.9%)	46 (5.3%)	<0.001	93 (4.5%)	27 (8.2%)	0.001
Comprehensive Community	2945 (31.6%)	213 (24.5%)		617 (29.8%)	88 (26.6%)	
Academic	3145 (33.8%)	411 (47.4%)		783 (37.8%)	129 (39.0%)	
Integrated Network	1834 (19.7%)	122 (14.1%)		396 (19.1%)	45 (13.6%)	
Unknown	935 (10.0%)	76 (8.8%)		182 (8.8%)	42 (12.7%)	
Insurance Status						
Uninsured	452 (4.9%)	34 (3.9%)	<0.001	174 (8.4%)	41 (12.4%)	0.005
Private Insurance	6955 (74.7%)	605 (69.7%)		1288 (62.2%)	192 (58.0%)	
Medicaid	1014 (10.9%)	102 (11.8%)		370 (17.9%)	62 (18.7%)	
Medicare	696 (7.5%)	101 (11.6%)		188 (9.1%)	20 (6.0%)	
Others/Unknown	198 (2.1%)	26 (3.0%)		51 (2.5%)	16 (4.8%)	

* Measures the significant changes observed in the outcome variable (surgery vs. NOM) in the different groups of each of the independent variables.

**Table 2:** Multivariable Cox proportional hazard model evaluating the impact of various variables on overall survival among patients with locally advanced rectal cancer.

Variable	T1-3 N+			T4 N+/−		
	Hazard Ratio	*p*-Value	95% Confidence Interval	Hazard Ratio	*p*-Value	95% Confidence Interval
Age (18–59 = ref)	1.01	0.005	1.00–1.01	1.01	0.104	1.00–1.02
Sex (Male = ref)						
Female	0.69	<0.001	0.62–0.77	0.84	0.041	0.72–0.99
Race (Non-Hispanic White = ref)						
Non-Hispanic Black	1.28	0.005	1.08–1.52	1.19	0.182	0.92–1.54
Asian	1.01	0.912	0.80–1.28	0.74	0.184	0.48–1.15
Hispanic	0.96	0.649	0.79–1.16	0.81	0.147	0.61–1.08
Others/Unknown	1.18	0.176	0.93–1.51	0.78	0.370	0.45–1.35
Charlson Comorbidity Index (0 = ref)						
1	1.43	<0.001	1.26–1.62	1.13	0.289	0.90–1.40
Surgery Status (Yes = ref)						
Non-operative Management	1.65	<0.001	1.40–1.94	2.73	<0.001	2.24–3.32
Insurance Status (Private = ref)			-			
Uninsured	1.77	<0.001	1.47–2.13	1.32	0.038	1.01–1.72
Medicaid	1.60	<0.001	1.38–1.86	1.25	0.040	1.01–1.56
Medicare	1.54	<0.001	1.31–1.81	1.26	0.122	0.94–1.68
Others/Unknown	1.32	0.077	0.97–1.80	1.64	0.012	1.11–2.43
Radiation Dose (≤5040 = ref)						
>5040	1.13	0.259	0.91–1.39	1.07	0.680	0.79–1.44

## Data Availability

The original contributions presented in the study are included in the article/Supplementary Material, further inquiries can be directed to the corresponding authors. This abstract was accepted for presentation at the Academic Surgical Congress in February 2024, Washington, DC, USA.

## Supplementary Materials

The following supporting information can be downloaded at https://www.mdpi.com/article/10.3390/cancers16122194/s1, Table S1: Results of propensity matching on a bias between the cohorts of patients in the National Cancer Database diagnosed with locally advanced rectal cancer between 2010–2020 undergoing surgery versus non-operative management (NOM). Figure S1: Effect of propensity matching on bias between the cohorts for patients in the National Cancer Database diagnosed with locally advanced rectal cancer undergoing surgery versus non-operative management (NOM) between 2010–2020 shown as Mahalonobis distance before and after propensity match.
