# Supplementary material for: Outcome of Patients with Locally Advanced Rectal Cancer Pursuing Non-Surgical Strategy in National Cancer Database
Source: Cancers (Basel). 2024 Jun 11;16(12):2194. doi: 10.3390/cancers16122194 (PMC11202149; doi:10.3390/cancers16122194)

Supplemental Figure S1: Effect of propensity matching on bias between the cohorts for patients in the National Cancer Database diagnosed with locally advanced rectal cancer undergoing surgery versus non-operative management (NOM) between 2010-2020 shown as Mahalonobis distance before and after propensity match.

T1-3 N+

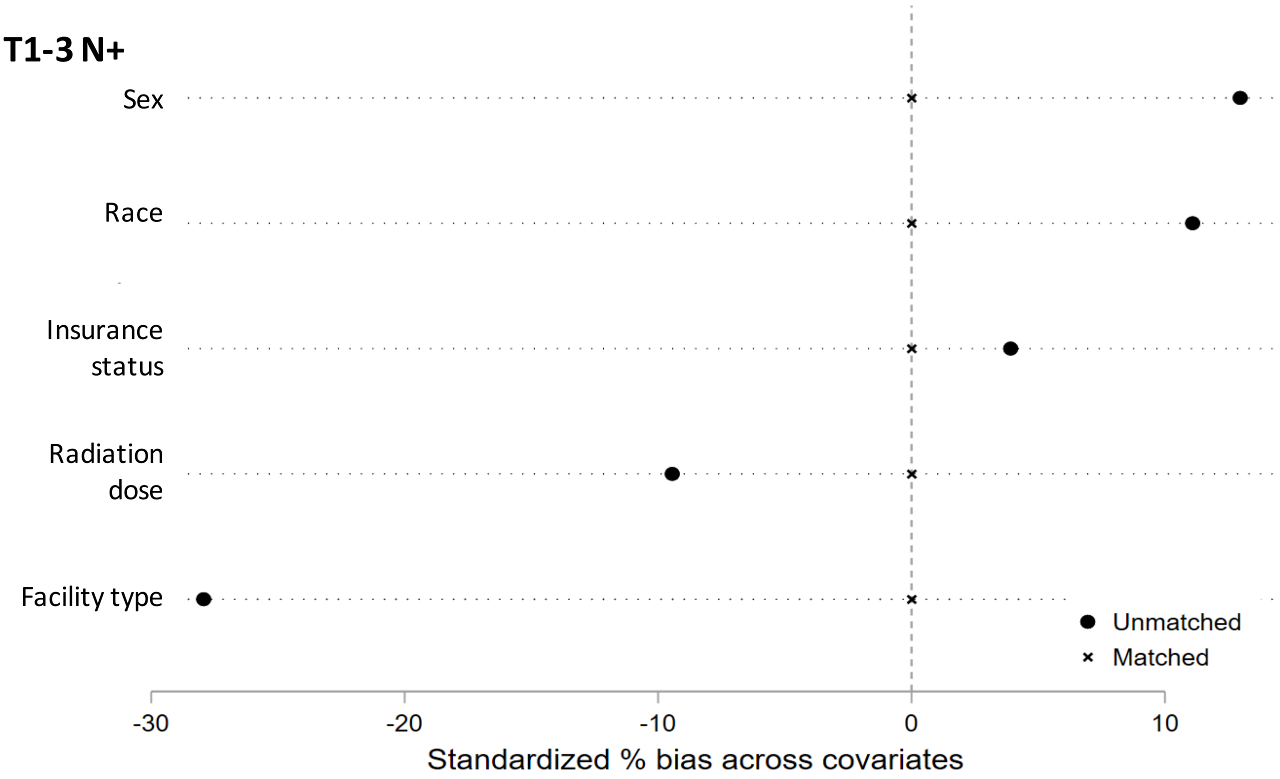

T4 N+/-

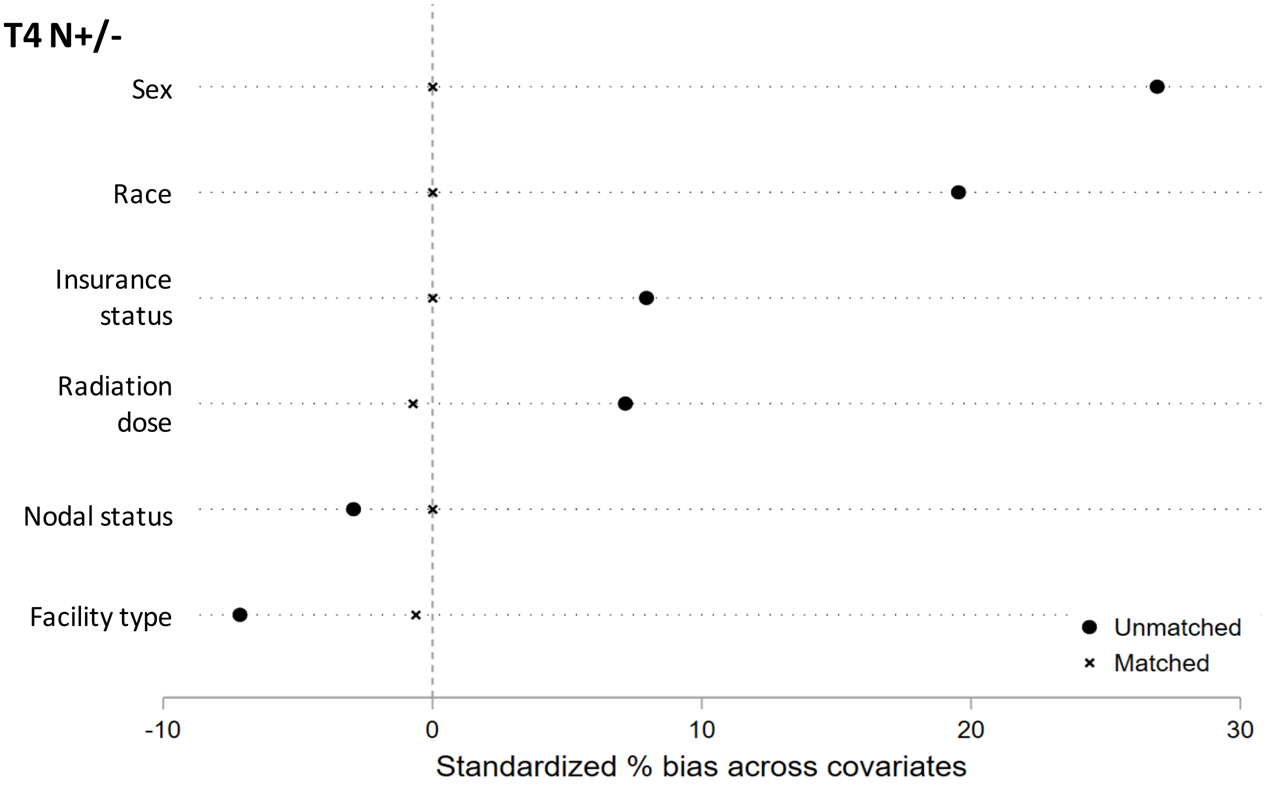

Supplement: Supplementary file 1 [file cancers-16-02194-s001.zip › Supplementary Figure 1.pdf]
