# Supplementary material for: Outcome of Patients with Locally Advanced Rectal Cancer Pursuing Non-Surgical Strategy in National Cancer Database
Source: Cancers (Basel). 2024 Jun 11;16(12):2194. doi: 10.3390/cancers16122194 (PMC11202149; doi:10.3390/cancers16122194)
Supplement: Supplementary file 1 [file cancers-16-02194-s001.zip › Supplementary Table 1.pdf]

Supplemental Table S1: Results of propensity matching on a bias between the cohorts of patients in the National Cancer Database diagnosed with locally advanced rectal cancer between 2010-2020 undergoing surgery versus non-operative management (NOM)

Variable		Mean		%Bias	p-value
		NOM	Surgery		
**T1-3 N+**					
**Sex**	Unmatched	33.1%	37.6%	-9.4	0.009
	Matched	33.1%	33.1%	0.0	1.000
**Race**	Unmatched	24.1%	22.4%	3.0	0.265
	Matched	24.1%	24.1%	0.0	1.000
**Insurance**	Unmatched	30.3%	25.3%	11.1	0.001
	Matched	30.3%	30.3%	0.0	1.000
**Radiation**	Unmatched	7.7%	4.6%	13.0	0.000
	Matched	7.7%	7.7%	0.0	1.000
**Facility type**	Unmatched	52.7%	66.2%	-27.9	0.000
		52.7%	52.7%	0.0	1.000
**T4 N+/-**					
**Sex**	Unmatched	39.7%	43.2%	-7.1	0.233
	Matched	39.7%	4.0%	-0.6	0.936
**Race**	Unmatched	37.2%	24.9%	26.9	<0.001
	Matched	37.2%	37.2%	0.0	1.000
**Insurance**	Unmatched	41.8%	38.0%	7.9	0.181
	Matched	41.8%	41.8%	0.0	1.000
**Radiation**	Unmatched	11.7%	6.1%	19.5	<0.001
	Matched	11.7%	11.7%	0.0	1.000
**Clinical nodal status**	Unmatched	78.2%	75.1%	7.2	0.238
	Matched	78.2%	78.5%	-0.7	0.924
**Facility type**	Unmatched	60.6%	62.0%	-2.9	0.622
	Matched	60.6%	60.6%	0.0	1.000
